# An Invitation from the Editors of *Cardio-Oncology*

**DOI:** 10.1186/s40959-015-0004-9

**Published:** 2015-11-26

**Authors:** Steven E. Lipshultz, Giorgio Minotti, Joseph Carver, Vivian I. Franco

**Affiliations:** 1 Department of Pediatrics, Wayne State University School of Medicine, the Children’s Research Center of Michigan, and the Children’s Hospital of Michigan, Detroit, MI USA; 2 Drug Sciences and Clinical Pharmacology, University Campus Bio-Medico, Rome, Italy; 3 Abramson Cancer Center of University of Pennsylvania Philadelphia, Philadelphia, PA USA

Advances in cancer treatments have brought hope to patients with these diseases once thought to be incurable. However, these same treatments can inadvertently harm healthy cells and affect systems unrelated to the cancer, especially the cardiovascular system [1]. A precise definition of cardiotoxicity is still lacking [2]. However, an overwhelming body of evidence has established that patients exposed to antitumor therapies have several laboratory or clinical indices of cardiovascular dysfunction and that become more evident as patients live longer. As a result, patients with cancer who have or who are at risk of cardiovascular toxicity are now being treated collaboratively by oncologists-hematologists, radiotherapists, and cardiologists, which has led to a new interdisciplinary field, cardio-oncology.

The journal *Cardio-Oncology* is a dedicated forum for oncologists, cardiologists, researchers, and other health providers who care for patients who have survived or who are being treated for cancer. It also provides the opportunity for the latest and highest quality evidence in this emerging field to be widely shared in the medical community. The mission of *Cardio-Oncology* is to publish research that addresses the balance between curing cancer and limiting the adverse cardiovascular effects of cancer treatment. To achieve this mission, we welcome a broad range of original research and review articles addressing many questions in this complex field from a multidisciplinary approach. Cardio-oncology has many areas of research. Here, we highlight some of the more pressing issues in the field and invite authors to submit for publication their work on these and other issues.

First, we have to better understand the risks of cardiovascular toxicity to establish effective prevention and surveillance protocols. A lack of awareness or underestimating the cardiovascular risks of cancer treatment among clinicians can inadvertently harm the patient in the long term. Many risk factors for cardiovascular toxicity have been identified in cancer patients during and after treatment. These factors include a cumulative dose of anthracycline (≥400 mg/m^2^ for adults [≥18 years old] and ≥300 mg/m^2^ for children [<18 years old]) [3], concomitant radiation therapy, younger or older age at diagnosis, female sex, black race, and the presence of other cardiovascular comorbidities.

However, the differences between patients who do and do not develop cardiotoxicity are substantial, even if they have some of the same risk factors. To explain these differences, investigators have explored possible genetic involvement. For example, hereditary hemochromatosis is a genetic disorder of iron metabolism that leads to iron overload-associated tissue injury. In this disorder, gene mutations in the C282Y allele are associated with myocardial injury in anthracycline-treated survivors of childhood acute lymphoblastic leukemia [4]. This mutation might cause excess iron accumulation in cardiac cells and increase the heart’s vulnerability to damage from the free radicals formed by the doxorubicin-iron complexes, but the actual mechanisms are not known. Another study found an increased risk of cardiomyopathy in patients exposed to low-to-moderate doses of anthracyclines. These patients were homozygous for the G allele in the carbonyl reductase 3 gene and presumably formed higher levels of a toxic anthracycline metabolite [5, 6]. Although additional studies are needed to validate these genetic risk factors, behavioral risks, such as smoking, physical inactivity, excess body weight, and alcohol consumption, also deserve further exploration and are becoming increasingly important as survivors increase in number and in age.

It is also necessary to understand the pathophysiology and mechanisms of cardiotoxicity of both old and new treatment regimens. Multiple mechanisms of cardiotoxicity have been proposed for anthracyclines, but less is known about other chemotherapeutics or the new generation of “targeted” drugs. Lessons from trastuzumab suggest that many new agents lack intrinsic severe cardiotoxicity but instead cause myocardial dysfunction and reduce cardiac defenses and repair mechanisms against anthracyclines or pathophysiologic stressors (e.g., hypertension) [7]. Other new drugs (antibodies, kinase inhibitors) may induce vasoconstriction, hypertension, or thrombosis by disrupting the nitric oxide pathway or other vasodilator mechanisms [7]. That said, cardiovascular toxicity from these newer agents seems to differ greatly from that of the anthracyclines. This difference is the basis for the simple but not all-inclusive classification of anthracyclines and “targeted” agents as type 1 and type 2 agents, respectively [8]. Clearly, much remains to be understood about these agents and their effects. This calls for preclinical models that identify risk and mechanisms of cardiotoxicity with a high level of predictability but unfortunately many cellular and animal models are limited by inherent pitfalls and do not always help decipher the cardiovascular toxic potential of one agent or another [9]. Newer approaches are much needed. Having clinicians and basic researchers work together may offer unprecedented opportunities to exchange information and provide mechanism-based information that translates into clinical facts.

Compared to the vast number of studies on cardiovascular toxicity and cancer in adults, the number of similar studies in children is almost sparse. Hence, many pediatric treatment protocols are extrapolated from those for adults, which is not always appropriate, given the differences in body composition and developmental changes in children. An example is the use of continuous anthracycline infusion, which reduces cardiotoxicity in adults. Limited evidence in children with high-risk acute lymphoblastic leukemia, however, indicates that continuous infusion is not more cardioprotective than bolus infusion of anthracyclines [10]. Hence, the lack of benefit is outweighed by the added expense of longer hospital stays and the increased risk of complications, suggesting that continuous infusion of anthracyclines be discontinued in children until there is more evidence to the contrary. Similarly, liposomal anthracyclines, given their unique pharmacokinetics, reduce the risk of cardiotoxicity in adults with solid tumors [11], but evidence of their effect in children is scarce and preliminary at best [12]. Also lacking are long-term follow-up studies to determine whether their use increases the risk of late cardiac effects.

In a similar manner, relatively few clinical studies include older cancer patients. In everyday clinical practice the elderly are often undertreated because of concerns about cardiovascular complications. This may lead to treatment modalities that lack curative effects, particularly when anthracyclines are administered in lower doses or fewer cycles or are replaced by less active drugs. Yet, there is evidence that full-dose anthracyclines could be safely administered to the elderly at risk for cardiotoxicity, provided that clinicians were expert enough to split the cumulative dose of anthracyclines in more numerous cycles as compared to standard protocols adopted in young-adult patients [13]. Again, this calls for newer forms of collaborations between oncologists, cardiologists and geriatricians, and for newer preclinical models in which cardiovascular liability of antitumor drugs in the elderly could be explored with a satisfactory level of predictability.

Addressing these issues may help inform treatment planning, which includes prevention and surveillance strategies. Altering cancer treatment to reduce or prevent toxic effects would be unethical without supporting evidence from well-designed studies. It is also important to ensure that preventive strategies do not interfere with oncologic efficacy or increase the risk of recurrence or secondary malignancy. Dexrazoxane, a multifunctional drug than can both chelate iron and inhibit topoisomerases, has important cardioprotective effects [14, 15] and is currently only approved by the FDA for use in adults with metastatic breast cancer who have received a cumulative dose of 300 mg/m^2^ of doxorubicin and who may benefit from continued treatment with an anthracycline [16]. Studies in children with high-risk cancer have also documented that dexrazoxane is cardioprotective when administered before each dose of doxorubicin [15], leading the FDA to designate dexrazoxane as a drug for orphan diseases in August 2014. Increasing evidence favors including dexrazoxane in anthracycline-based treatment protocols [17]. However, the cardioprotection afforded by dexrazoxane has been limited to patients receiving anthracyclines.

Another important factor related to cardioprotection is the timing of administering or implementing these treatments. For example, in adults, dexrazoxane administration is recommended only after a patient has already received 300 mg/m^2^ of doxorubicin to protect the heart from any subsequent exposure to anthracycline. In contrast, to prevent irreversible cardiac damage to the developing heart, dexrazoxane is more effective in children if given as first-line therapy before each dose of doxorubicin. In this case, the reason for this difference in timing is understandable – children are more vulnerable to cardiac damage than adults because their hearts are still developing. However, the reasons for other differences are not so clear. Enalapril is often given after cancer therapy to treat cardiac damage that manifest later in follow up, but some studies suggest that cardiovascular drugs could be given during treatment to protect the heart [18]. It has also been shown that the earlier that heart failure treatment is started after biomarker evidence of cardiac damage/dysfunction, the less likely is the development of late myocardial dysfunction [19]. In childhood cancer survivors with asymptomatic and symptomatic cardiac dysfunction treated with enalapril after completing chemotherapy, left ventricular structure and function improved temporarily, but after 6 to 10 years of enalapril treatment, these improvements were lost, and in some cases were even reversed [20].

On the other hand, in a randomized trial of adults with hematologic malignancies, patients received either combination cardiac therapy with enalapril and carvedilol (intervention) or no additional treatment (controls) during chemotherapy [18]. After 6 months, left ventricular ejection fraction was significantly reduced in the control group but was unchanged in the intervention group. Although the results of this short-term follow-up are promising, whether the benefits will be lost as it does in childhood cancer survivors is unknown.

Surveillance of cardiotoxicity is most commonly done using echocardiographic assessment of left ventricular ejection fraction. However, echocardiography is limited by preload and unavoidable operator-dependent biases. Further, it may not always detect cardiac abnormalities that precede decrements in ejection fraction and that require early treatment with cardiovascular drugs. Other promising methods include cardiac MRI, which can be cost-prohibitive, and strain and strain rate echocardiography, which require additional studies to validate their results.

Serum cardiac biomarkers are commonly used to assess cardiac status in patients without cancer, and they may detect early cardiac damage in children. In survivors of childhood high-risk acute lymphoblastic leukemia, elevated concentrations of cardiac troponin T (cTnT) and N-terminal probrain natriuretic peptide (NT-proBNP) during the first 90 days of treatment were associated with abnormal echocardiographic findings 4 years later, suggesting that these biomarkers might help predict early cardiac damage [21]. In adults, elevated concentrations of troponin [22] and NT-proBNP [23] preceded decrements in left ventricular ejection fraction. However, additional studies are needed before these markers can inform treatment decisions in daily practice.

## Future directions

It is becoming clear that individual risks alone do not predict cardiotoxicity, which suggests that specific risk groups might be identified and studied for the cardiovascular effects of tailored treatment protocols. Understanding the mechanisms of treatment-related cardiotoxicity might help identify potential areas of drug development. It is also important to continue exploring a variety of cardioprotective strategies in studies carefully designed to reliably measure and compare their efficacy and safety. Lastly, it may be possible to establish evidence-based guidelines to inform treatment decisions unique to each patient to ensure the proper balance of care.

We have mentioned only a few unsettled issues in cardio-oncology. Many other questions await answers and clinical directions, and many more will emerge as oncologists, hematologists, radiation therapists, and pharmacologists test new drugs and treatment protocols. We trust that this new journal, *Cardio-Oncology*, will become a valuable forum for presenting preclinical and clinical research on this complex field of medicine. The Editors welcome suggestions and encourage all cardio-oncologists to submit their work to *Cardio-Oncology*. As is the case for all journals, the success of *Cardio-Oncology* depends on the enthusiasm, the scientific curiosity, and the support the scientific and clinical community and their authors.

## References

[1] Carver JR, Shapiro CL, Ng A, Jacobs L, Schwartz C, Virgo KS (2007). American Society of Clinical Oncology clinical evidence review on the ongoing care of adult cancer survivors: cardiac and pulmonary late effects. J Clin Oncol.

[2] Yeh ET, Salvatorelli E, Menna P, Minotti G (2014). What is cardiotoxicity?. Prog Pediat Cardiol.

[3] Children’s Oncology Group. Long-term follow-up guidelines for survivors of childhood, adolescent, and young adult cancers, version 4.0., Oct. 2013. Available at: http://www.survivorshipguidelines.org/pdf/LTFUGuidelines_40.pdf; Accessed: March 23, 2015.

[4] Lipshultz SE, Lipsitz SR, Kutok JL, Miller TL, Colan SD, Neuberg DS (2013). Impact of hemochromatosis gene mutations on cardiac status in doxorubicin-treated survivors of childhood high-risk leukemia. Cancer.

[5] Blanco JG, Sun CL, Landier W, Chen L, Esparza-Duran D, Leisenring W (2012). Anthracycline-related cardiomyopathy after childhood cancer: role of polymorphisms in carbonyl reductase genes--a report from the Children’s Oncology Group. J Clin Oncol.

[6] Minotti G, Salvatorelli E, Menna P (2010). Pharmacological foundations of cardio-oncology. J Pharmacol Exp Ther.

[7] Salvatorelli E, Menna P, Cantalupo E, Chello M, Covino E, Wolf FI, et al. The concomitant management of cancer therapy and cardiac therapy. Biochim Biophys Acta. 2015; doi: 10.1016/j.bbamem.2015.01.003. [Epub ahead of print] 10.1016/j.bbamem.2015.01.003 25596534

[8] Suter TM, Ewer MS (2013). Cancer drugs and the heart: importance and management. Eur Heart J.

[9] Menna P, Salvatorelli E, Minotti G (2008). Cardiotoxicity of antitumor drugs. Chem Res Toxicol.

[10] Lipshultz SE, Miller TL, Lipsitz SR, Neuberg DS, Dahlberg SE, Colan SD (2012). Continuous versus bolus infusion of doxorubicin in children with ALL: long-term cardiac outcomes. Pediatrics.

[11] Van Dalen EC, Michiels EM, Caron HN, Kremer LC (2010). Different anthracycline derivates for reducing cardiotoxicity in cancer patients. Cochrane Database Syst Rev.

[12] Sieswerda E, Kremer LC, Caron HN, Van Dalen EC (2011). The use of liposomal anthracycline analogues for childhood malignancies: A systematic review. Eur J Cancer.

[13] Carver JR, Schuster SJ, Glick JH (2008). Doxorubicin cardiotoxicity in the elderly: old drugs and new opportunities. J Clin Oncol.

[14] Van Dalen EC, Caron HN, Dickinson HO, Kremer LC (2011). Cardioprotective interventions for cancer patients receiving anthracyclines. Cochrane Database Syst Rev.

[15] Lipshultz SE, Scully RE, Lipsitz SR, Sallan SE, Silverman LB, Miller TL (2010). Assessment of dexrazoxane as a cardioprotectant in doxorubicin-treated children with high-risk acute lymphoblastic leukaemia: long-term follow-up of a prospective, randomised, multicentre trial. Lancet Oncol.

[16] Minotti G, Menna P, Salvatorelli E, Cairo G, Gianni L (2004). Anthracyclines: molecular advances and pharmacologic developments in antitumor activity and cardiotoxicity. Pharmacol Rev.

[17] Lipshultz SE, Franco VI, Sallan SE, Adamson PC, KSteiner R, Swain SM (2014). Dexrazoxane for reducing anthracycline-related cardiotoxicity in children with cancer: An update of the evidence. Prog Pediatr Cardiol.

[18] Bosch X, Rovira M, Sitges M, Domenech A, Ortiz-Perez JT, De Caralt TM (2013). Enalapril and carvedilol for preventing chemotherapy-induced left ventricular systolic dysfunction in patients with malignant hemopathies: the OVERCOME trial (preventiOn of left Ventricular dysfunction with Enalapril and caRvedilol in patients submitted to intensive ChemOtherapy for the treatment of Malignant hEmopathies). J Am Coll Cardiol.

[19] Cardinale D, Colombo A, Sandri MT, Lamantia G, Colombo N, Civelli M (2006). Prevention of high-dose chemotherapy–induced cardiotoxicity in high-risk patients by angiotensin-converting enzyme inhibition. Circulation.

[20] Lipshultz SE, Lipsitz SR, Sallan SE, Simbre VC, Shaikh SL, Mone SM (2002). Long-term enalapril therapy for left ventricular dysfunction in doxorubicin-treated survivors of childhood cancer. J Clin Oncol.

[21] Lipshultz SE, Miller TL, Scully RE, Lipsitz SR, Rifai N, Silverman LB (2012). Changes in cardiac biomarkers during doxorubicin treatment of pediatric patients with high-risk acute lymphoblastic leukemia: associations with long-term echocardiographic outcomes. J Clin Oncol.

[22] Cardinale D, Sandri MT, Martinoni A, Tricca A, Civelli M, Lamantia G (2000). Left ventricular dysfunction predicted by early troponin I release after high-dose chemotherapy. J Am Coll Cardiol.

[23] Romano S, Fratini S, Ricevuto E, Procaccini V, Stifano G, Mancini M (2011). Serial measurements of NT-proBNP are predictive of not-high-dose anthracycline cardiotoxicity in breast cancer patients. Br J Cancer.

